# Effect of a Positive Psychological Intervention on Pain and Functional Difficulty Among Adults With Osteoarthritis: A Randomized Clinical Trial

**DOI:** 10.1001/jamanetworkopen.2018.2533

**Published:** 2018-09-21

**Authors:** Leslie R. M. Hausmann, Ada Youk, C. Kent Kwoh, Rollin M. Gallagher, Debra K. Weiner, Ernest R. Vina, D. Scott Obrosky, Genna T. Mauro, Shauna McInnes, Said A. Ibrahim

**Affiliations:** 1 Center for Health Equity Research and Promotion, Veterans Affairs Pittsburgh Healthcare System, Pittsburgh, Pennsylvania; 2 School of Medicine, University of Pittsburgh, Pittsburgh, Pennsylvania; 3 Graduate School of Public Health, University of Pittsburgh, Pittsburgh, Pennsylvania; 4 University of Arizona Arthritis Center, University of Arizona, Tucson; 5 College of Medicine, University of Arizona, Tucson; 6 Center for Health Equity Research and Promotion, Corporal Michael J. Crescenz Veterans Affairs Medical Center, Philadelphia, Pennsylvania; 7 School of Medicine, University of Pennsylvania, Philadelphia; 8 Geriatric Research, Education, and Clinical Center, Veterans Affairs Pittsburgh Healthcare System, Pittsburgh, Pennsylvania; 9 Department of Healthcare Policy and Research, Weill Cornell Medicine, New York, New York

## Abstract

**Importance:**

Positive psychological interventions for improving health have received increasing attention recently. Evidence on the impact of such interventions on pain, and racial disparities in pain, is limited.

**Objective:**

To assess the effects of a positive psychological intervention on pain and functional difficulty in veterans with knee osteoarthritis.

**Design, Setting, and Participants:**

The Staying Positive With Arthritis Study is a large, double-blinded randomized clinical trial powered to detect race differences in self-reported pain in response to a positive psychological intervention compared with a neutral control intervention. Data were collected from 2 urban Veterans Affairs medical centers. Participants included non-Hispanic white and non-Hispanic African American patients aged 50 years or older with a diagnosis of osteoarthritis. Mailings were sent to 5111 patients meeting these criteria, of whom 839 were fully screened, 488 were eligible, and 360 were randomized. Enrollment lasted from July 8, 2015, to February 1, 2017, with follow-up through September 6, 2017.

**Interventions:**

The intervention comprised a 6-week series of evidence-based activities to build positive psychological skills (eg, gratitude and kindness). The control program comprised similarly structured neutral activities. Programs were delivered via workbook and weekly telephone calls with interventionists.

**Main Outcomes and Measures:**

The primary outcomes were self-reported pain and functional difficulty measured using the Western Ontario and McMaster Universities Osteoarthritis Index (WOMAC; range 0-100). Secondary outcomes included affect balance and life satisfaction.

**Results:**

The sample included 180 non-Hispanic white patients and 180 non-Hispanic African American patients (mean [SD] age, 64.2 [8.8] years; 76.4% were male). Mean (SD) baseline scores for WOMAC pain and functional difficulty were 48.8 (17.6) and 46.8 (18.1), respectively. Although both decreased significantly over time (pain: χ^2^_3_ = 49.50, *P* < .001; functional difficulty: χ^2^_3_ = 22.11, *P* < .001), differences were small and did not vary by treatment group or race. Exploratory analyses suggested that the intervention had counterintuitive effects on secondary outcomes.

**Conclusions and Relevance:**

The results of this randomized clinical trial do not support the use of positive psychological interventions as a stand-alone treatment for pain among white or African American veterans with knee osteoarthritis. Adaptations are needed to identify intervention components that resonate with this population, and the additive effect of incorporating positive psychological interventions into more comprehensive pain treatment regimens should be considered.

**Trial Registration:**

ClinicalTrials.gov Identifier: NCT02223858

## Key Points

**Question:**

Is positive psychology effective as a treatment for chronic arthritis pain and does it reduce race disparities in pain management?

**Findings:**

In this randomized clinical trial involving 360 Veterans Affairs patients with chronic pain from osteoarthritis, a 6-week telephone-administered positive psychological intervention did not improve pain or functional difficulty vs a control program. No difference by race was found in the effect of the intervention.

**Meaning:**

A telephone-administered positive psychological intervention was not associated with improvement in chronic pain or functional difficulty from osteoarthritis for either white or African American patients.

## Teaser

This randomized clinical trial assesses the effects of a positive psychological intervention on pain and functional difficulty vs a control program in veterans with knee osteoarthritis and evaluates whether this management varies by race.

## Introduction

With increasing acceptance of complementary and integrative health practices, there has been a surge of interest in using positive psychological interventions to improve the well-being of patients with chronic illness.^1,2,3,4,5,6,7^ Such interventions include activities that increase positive affect and cultivate qualities such as gratitude and kindness,^1,2,8,9^ and are based on theoretical and empirical work linking positive psychological skills and health.^1,8,10^ Evidence indicates that positive psychological interventions reduce depressive symptoms and increase overall well-being.^1,2,3^ Studies have begun testing the effects of positive psychological interventions in patient populations with chronic health conditions other than depression,^11,12,13,14,15,16,17,18,19^ and have started examining their effects on physical outcomes such as pain.^18,20,21,22,23,24^

The potential of positive psychological interventions to relieve chronic pain is supported by work demonstrating that positive affect can promote pain resiliency through neurobiological and cognitive pathways.^25^ Although some evidence suggests that participating in a positive psychological intervention decreases pain,^18,21,24^ reviews of extant research have concluded that large, well-controlled randomized trials are needed to delineate the benefits and limitations of positive psychological interventions for use in clinical care.^3,4,5^

This article reports findings of the Staying Positive With Arthritis Study, the largest randomized clinical trial, to our knowledge, testing the effects of a positive psychological intervention on self-reported pain and functional difficulty in patients with chronic pain from knee osteoarthritis (OA).^26^ The most common form of arthritis,^27^ OA is a condition for which positive psychological interventions have not previously been tested in a large trial. The objective of the Staying Positive With Arthritis Study was to evaluate the effect of a positive psychological intervention, compared with an active control program, on pain and functional difficulty in a predominantly male sample of non-Hispanic white and non-Hispanic African American patients with knee OA. We hypothesized that patients randomized to a 6-week positive psychological intervention (vs control program) would report greater improvements in the primary outcomes of self-reported pain and functional difficulty from baseline to 6 months, and that improvements would be larger for African American patients than for white patients. We powered the study to detect racial differences in response to the intervention because African American individuals (vs white individuals) tend to report worse OA-related pain and disability^28,29,30^ and express stronger preferences for nontraditional, nonpharmacological approaches to pain management.^31,32,33,34,35^ Affect balance^36,37^ and life satisfaction^38^ were secondary outcomes.

## Method

### Study Participants and Recruitment Strategy

The full study protocol and statistical analysis plan are published elsewhere^26^ and are available in Supplement 1. Briefly, patients with symptomatic knee OA from Veterans Affairs (VA) medical centers in Pittsburgh and Philadelphia, Pennsylvania, were recruited by mail and telephone. Mailings were sent to patients meeting basic eligibility criteria based on their VA medical records (Table 1). Patients who expressed interest or did not respond within 2 weeks were telephoned to be fully screened for eligibility (Table 1).^39,40^ Patients who learned about the study from flyers at participating sites were also screened. The VA Central institutional review board approved the study. We followed the Consolidated Standards of Reporting Trials (CONSORT) reporting guideline

**Table 1.** Staying Positive With Arthritis Study Inclusion and Exclusion Criteria for Initial Mailing and Full Study^a^

Inclusion	Exclusion
Eligibility criteria for initial mailing (based on VA electronic medical record)	
Aged ≥50 y	Deceased
Non-Hispanic white or non-Hispanic African American race	Nonveteran
Had a primary care appointment at a participating site in the past 12 mo	Inflammatory arthritis (rheumatoid arthritis [*ICD-9*: 714.xx], lupus [*ICD-9*: 695.4, 710.0], psoriatic arthritis [*ICD-9*: 696.0], and ankylosing spondylitis [*ICD-9*: 720.0])
Osteoarthritis (*ICD-9*: 715)	Alzheimer disease and dementia (*ICD-*9: 294.xx, 290.xx, 291.xx, 331.xx, 094.1)
Eligibility criteria for enrollment (based on telephone screen)	
Aged ≥50 y	Self-reported serious problems with hearing, eyesight, or memory
Non-Hispanic white or non-Hispanic African American race	Diagnosed with any type of arthritis other than osteoarthritis or degenerative arthritis
Receives primary care at a participating site	Treated for cancer in the last 3 y
Frequent pain characteristic of symptomatic knee osteoarthritis^39^	Had a steroid injection for knee pain in the past 3 mo
Pain in worst knee during the past wk rated as ≥4 on a 0-10 scale	Had a knee replacement in the past 3 mo
Speak, read, and write in English	Plan to have a knee replacement in the next 6 mo
	Self-reported inability to complete study-related telephone calls and program activities that involve reading and writing
	No reliable telephone number
	Answering ≥2 items incorrectly on a 6-item screener for cognitive impairment^40^

Abbreviations: *ICD-9*, *International Classification of Diseases, Ninth Revision*; VA, Veterans Affairs.

^a^ Eligibility was determined based on self-reported responses to a screening survey.

### Study Protocol

Eligible patients attended an in-person baseline visit where they provided written informed consent, completed a staff-administered baseline assessment, and were randomized to a 6-week positive psychological intervention or neutral control program. Randomization was at the patient level, stratified by study site, and patient race (non-Hispanic white or non-Hispanic African American), with a 1 to 1 allocation using random block sizes of 2, 4, 6, or 8. The statistician placed positive and control program workbooks in sealed envelopes according to the randomization sequence and the staff took the next sealed envelope to each baseline visit. After collecting baseline measures, staff opened the envelope, oriented participants to their workbook, and reviewed the first activity, which participants completed over the next week. The staff called participants weekly for the next 6 weeks to assess adherence and review the next activity. Outcomes were collected via telephone surveys 1, 3, and 6 months after the final week. Participants were compensated up to $110. Patients and staff who collected baseline and outcome measures were blinded to the treatment group.

### Intervention

The intervention was an individually based program, in which participants completed 1 new positive psychological activity for the first 5 weeks and repeated their favorite in week 6.^26^ Activities, which were adapted for the target population,^24^ included recalling and reflecting on positive events^1,41^; writing a letter of gratitude^1,42^; cultivating mindfulness^43,44^; practicing kindness^45^; and increasing engagement in activities that they enjoy, give them a sense of achievement, or bring them closer to others (a variant of behavioral activation).^9^

### Control Program

The control program was identical to the intervention in terms of framing, reading level, format, duration, and delivery, but contained neutral control activities adapted from previous positive psychological intervention studies.^1,42,46,47^ Control activities asked participants to recall events that affected them each day, identify ways they could change their life circumstances, recall early memories, record things they did in the past week, plan their day, and repeat their favorite activity in week 6.

### Intervention Delivery and Fidelity

Staff was trained to deliver the intervention and control program in a 1.5-day workshop co-led by the principal investigator and a positive psychologist. Prior to delivering the programs to participants, staff demonstrated proficiency in delivering both programs in calls with the positive psychologist. Staff participated in frequent cross-site calls to ensure uniform delivery of the program throughout enrollment (July 8, 2015, through February 1, 2017).

### Study Measures

#### Primary Outcomes: Osteoarthritis Pain and Functional Difficulty

Primary outcomes included pain (5 items) and physical function (17 items) subscales of the Western Ontario and McMaster Universities Osteoarthritis Index (WOMAC).^48,49^ Subscale scores were calculated as the sum of items, then transformed to a 0 to 100 scale (higher = worse).

#### Secondary Outcomes: Affect Balance and Life Satisfaction

Well-being measures commonly used in positive psychological intervention studies were included as secondary outcomes. Affect balance was assessed using the International Positive and Negative Affect Schedule Short Form, which asks how often participants felt 5 positive (eg, inspired) or 5 negative (eg, upset) emotions over the past week (1 = never; 5 = always).^50^ Affect balance was calculated by subtracting the sum of negative scores from the sum of positive scores.^36,37^ Life satisfaction was assessed using a 5-item scale that asked participants the extent to which they agreed with statements such as “In most ways, your life is close to your ideal” (1 = strongly disagree; 5 = strongly agree).^38^

#### Demographic and Clinical Characteristics

Race and ethnicity categorization was based on self-reported responses to the following questions: are you of Spanish, Hispanic, or Latino origin (including Mexican, Puerto Rican, Cuban, South or Central American, or other Spanish culture or origin)? (yes or no); and which category best describes your race: white, black or African American, Asian, Native Hawaiian or other Pacific Islander, American Indian or Alaska Native, or other (check all that apply)? Additional baseline characteristics included self-reported sex, age, income, education, employment, marital status, general health status, health literacy,^51,52^ physical comorbid medical conditions,^53^ and past diagnoses and current treatment of depression or anxiety.^54^ Patients were asked to report whether they were using several pharmacological and nonpharmacological OA treatments assessed in the Osteoarthritis Initiative.^39^ Body mass index and whether participants had radiography or magnetic resonance imaging reports documenting radiographic evidence of OA were ascertained from VA medical records.

#### Intervention Adherence and Engagement

In weekly telephone calls during the intervention period, participants were asked to recall the activity they were supposed to complete the previous week and to indicate whether they completed it entirely, partially, or not at all.^6^ Adherence was calculated as the number of weekly calls completed and the number of correctly identified activities that were partially or entirely completed.

Participants who reported at least partially completing an activity were also asked to rate the benefit, enjoyment, and difficulty of each exercise using a 7-point scale (1 = not at all; 7 = extremely).^6^ These ratings were treated as continuous indicators of intervention engagement.

### Statistical Analysis

Analyses were performed using Stata, version 14 (Stata Corp).^55^ We checked outcome measures for normality and found no violations. Descriptive statistics were computed as means and standard deviations for continuous variables and frequencies and percentages for categorical variables. Time was treated as a 4-level categorical variable based on plots showing a nonlinear change over time in the unadjusted primary outcomes. All models were adjusted for study site. Statistical significance was determined as *P* < .05 and all tests were 2-sided.

We compared intervention adherence across treatment and racial groups using separate logistic regression models testing the main effects of treatment group and participant race, and a model testing the treatment group × race interaction (including the main effects), on 2 binary adherence measures (completed ≥5 calls; entirely or partially completed ≥5 correct activities). We used linear mixed models to test the same effects for the repeated measures of intervention engagement.

We tested study hypotheses using linear mixed models that allowed the use of data from participants with missing data from 1 or more time points. In separate models for each outcome, we included fixed effects for treatment group, race, time, and all 2-way and 3-way interactions. We tested the statistical significance of the main effects and interaction terms using likelihood ratio tests via χ^2^ statistics. When the 3-way interaction was not significant, terms with race were removed and the treatment group × time interaction was examined. When this interaction was not significant, a model testing only the simple main effect of time was examined. We tested the study hypotheses using intention-to-treat analyses.^56^ We tested models adjusting only for site and models adjusting for all baseline characteristics. We also conducted exploratory subgroup analyses to elucidate our primary findings.

#### Missing Data and Power

For scales with 20% or fewer missing items, missing items were replaced with the mean of the remaining items. Scales with more than 20% missing items were treated as missing. A sample size of 360 patients (180 non-Hispanic white and 180 non-Hispanic African American) was chosen based on a priori power calculations to detect a 3-way interaction between treatment group, race, and time, assuming a 20% change in baseline, the WOMAC pain subscale scores, and 80% power.^26^

## Results

### Baseline Sample Characteristics

**Figure 1.**
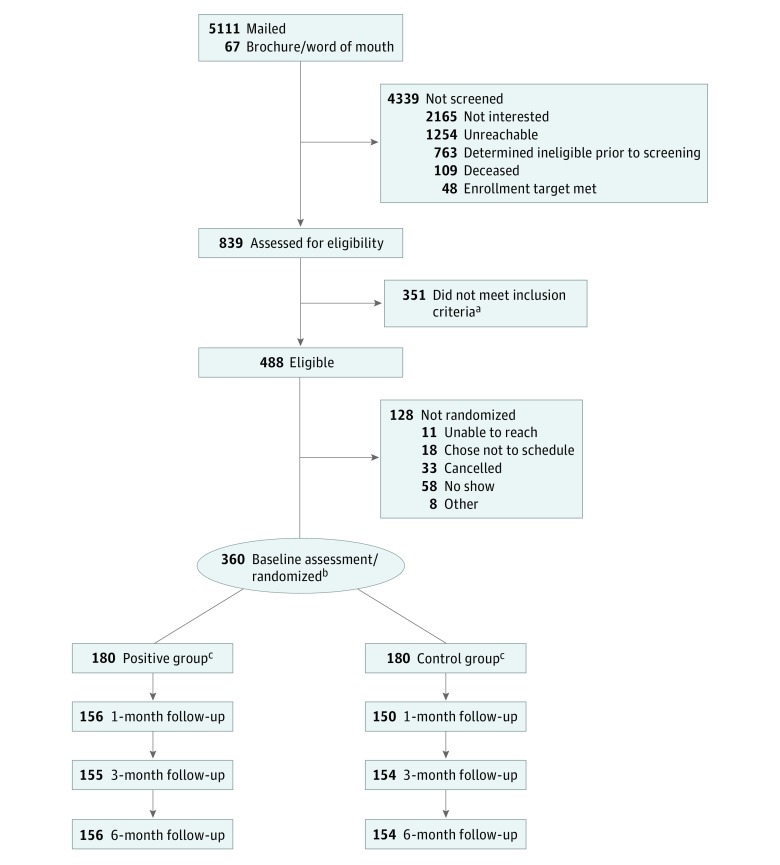
CONSORT Flow Diagram for the Staying Positive With Arthritis Study ^a^Reasons for ineligibility are provided in eTable 1 in Supplement 2. ^b^Randomization was at the patient level, stratified by site and self-reported race. ^c^Details on reasons for missing data points are provided in eTable 2 and eTable 3 in Supplement 2.

Of 5111 patients who were sent mailings and 67 who responded to study brochures, 839 completed the full telephone screening, 488 were eligible, and 360 were enrolled and randomized (Figure 1; eTables 1-3 in Supplement 2). Participants included 180 non-Hispanic white and 180 non-Hispanic African American patients (mean [SD] age, 64.2 [8.8] years; 76.4% were male) (Table 2). The mean (SD) pain rating at screening was 7.2 (1.7) on a scale of 0 to 10, and 63.6% of participants had an x-ray or MRI indicating OA in their VA medical record. There were several differences between African American and white patients (Table 2). For example, compared with white patients, African American patients were less likely to be married or living with a partner (99 [55.0%] vs 65 [36.1%]), more likely to be disabled or unemployed (50 [27.8%] vs 76 [42.2%]), and less likely to have a college degree (54 [30%] vs 36 [20%]).

**Table 2.** Baseline Characteristics by Treatment Group and Race

Variable	No. (%)				
	Total (N = 360)	Treatment Group		Participant Race	
		Positive (n = 180)	Control (n = 180)	White (n = 180)	African American (n = 180)
Age, mean (SD), y	64.2 (8.8)	64.4 (9.4)	64.1 (8.1)	65.9 (9.2)	62.6 (8.0)
BMI, mean (SD)	31.8 (6.5)	31.8 (6.4)	31.9 (6.6)	32.4 (6.7)	31.3 (6.3)
Female	85 (23.6)	44 (24.4)	41 (22.8)	42 (23.3)	43 (23.9)
Site					
Site A	180 (50.0)	90 (50.0)	90 (50.0)	90 (50.0)	90 (50.0)
Site B	180 (50.0)	90 (50.0)	90 (50.0)	90 (50.0)	90 (50.0)
Married or living with partner	164 (45.6)	80 (44.4)	84 (46.7)	99 (55.0)	65 (36.1)
Employment status					
Employed	88 (24.4)	46 (25.6)	42 (23.3)	38 (21.1)	50 (27.8)
Retired	146 (40.6)	72 (40.0)	74 (41.1)	92 (51.1)	54 (30.0)
Disabled/unemployed/other	126 (35.0)	62 (34.4)	64 (35.6)	50 (27.8)	76 (42.2)
Income, $					
<20 000	103 (28.6)	48 (26.7)	55 (30.6)	36 (20.0)	67 (37.2)
20 000-39 999	100 (27.8)	52 (28.9)	48 (26.7)	44 (24.4)	56 (31.1)
≥40 000	136 (37.8)	67 (37.2)	69 (38.3)	88 (48.9)	48 (26.7)
Do not know/refused	21 (5.8)	13 (7.2)	8 (4.4)	12 (6.7)	9.0 (5.0)
Education					
≤High school	109 (30.3)	56 (31.1)	53 (29.4)	49 (27.2)	60 (33.3)
Some college	161 (44.7)	75 (41.7)	86 (47.8)	77 (42.8)	84 (46.7)
≥4 y degree	90 (25.0)	49 (27.2)	41 (22.8)	54 (30.0)	36 (20.0)
Adequate health literacy	284 (78.9)	138 (76.7)	146 (81.1)	145 (80.6)	139 (77.2)
Good, very good, or excellent self-rated health	218 (60.6)	108 (60.0)	110 (61.1)	124 (68.9)	94 (52.2)
Charlson comorbidity index (self-report)					
0-1	104 (28.9)	52 (28.9)	52 (28.9)	49 (27.2)	55 (30.6)
2-3	121 (33.6)	61 (33.9)	60 (33.3)	55 (30.6)	66 (36.7)
≥4	135 (37.5)	67 (37.2)	68 (37.8)	76 (42.2)	59 (32.8)
Pain rating on 0-10 scale, mean (SD)	7.2 (1.7)	7.2 (1.6)	7.3 (1.7)	6.9 (1.7)	7.6 (1.5)
Anxiety disorder (self-report)	141 (39.2)	66 (36.7)	75 (41.7)	72 (40.0)	69 (38.3)
Depressive disorder (self-report)	166 (46.1)	78 (43.3)	88 (48.9)	78 (43.3)	88 (48.9)
Being treated for mental health or emotional condition (self-report)	122 (33.9)	51 (28.3)	71 (39.4)	64 (35.6)	58 (32.2)
No. of treatments currently being used for joint paint or arthritis, mean (SD)					
Pharmacological (possible range: 0-6)^a^	1.6 (1.0)	1.5 (1.0)	1.6 (1.1)	1.6 (1.0)	1.6 (1.1)
Nonpharmacological or alternative (possible range: 0-13)^b^	3.1 (2.0)	3.1 (2.0)	3.1 (2.0)	3.2 (2.1)	3.1 (1.9)
Radiographic evidence of OA^c^					
No x-ray or MRI on file	115 (31.9)	57 (31.7)	58 (32.2)	64 (35.6)	51 (28.3)
X-ray or MRI with no indication of OA	16 (4.4)	8 (4.4)	8 (4.4)	9 (5.0)	7 (3.9)
X-ray or MRI on file with indication of OA	229 (63.6)	115 (63.9)	114 (63.3)	107 (59.4)	122 (67.8)

Abbreviations: BMI, body mass index (calculated as weight in kilograms divided by height in meters squared); MRI, magnetic resonance imaging; OA, osteoarthritis.

^a^ Count of the following treatments reportedly being used at baseline: acetaminophen, nonsteroidal anti-inflammatory drugs, topical nonsteroidal anti-inflammatory drugs, cyclooxygenase-2 selective inhibitors, opioids, and hyaluronic acid or steroid injections.

^b^ Count of the following treatments reportedly being used at baseline: acupuncture, acupressure, or massage therapy; chiropractic care; homeopathy or naturopathy; physical therapy; water- or land-based exercise; health supplements for joint pain; vitamins; herbs; topical creams or oils; copper bracelets or magnets; yoga, tai chi, chi gong, pilates; relaxation or mind-body activities; and spiritual activities.

^c^ No x-ray or MRI on file and x-ray or MRI with no indication of OA were combined to create a dichotomous indicator of radiographic evidence of OA (no or yes) for analyses.

### Adherence and Engagement

During the 6-week intervention, 287 participants (79.7%) completed 5 or more weekly calls, and 234 participants (65.0%) reported entirely or partially completing 5 or more correct activities. Adherence rates did not significantly differ by treatment group or race (*P* > .05; eTable 4 in Supplement 2). The positive (vs control) group rated the weekly activities as more beneficial (mean [SD]: 5.77 [1.32] vs 5.39 [1.68]; *P* = .001) and more enjoyable (mean [SD]: 5.91 [1.30] vs 5.33 [1.72]; *P* < .001), but as equally difficult (mean [SD]: 2.26 [1.80] vs 2.23 [1.87]; *P* = .95). Ratings did not differ by participant race (eTable 5 in Supplement 2).

### Primary Outcomes: Pain and Functional Difficulty

Participants at baseline reported mean (SD) WOMAC pain and functional difficulty scores of 48.8 (17.6) and 46.8 (18.1), respectively. The hypothesized 3-way interaction between treatment group, race, and time was not significant for either outcome (Table 3). Models omitting nonsignificant interactions revealed no interactions between treatment group and time. Pain and functional difficulty both decreased significantly over time (pain, mean: 48.8 at baseline, 44.5 at 1 month, 43.6, at 3 months, and 42.4 at 6 months; overall test for time: χ^2^_3_ = 49.50, *P* < .001; functional difficulty, mean: 46.8 at baseline, 43.9 at 1 month, 43.4 at 3 months, and 42.9 at 6 months; overall test for time: χ^2^_3_ = 22.11, *P* < .001). Results were similar in models fully adjusting for all baseline characteristics.

**Table 3.** Change in Self-reported Pain and Functional Difficulty in White and African American Patients With Knee or Hip Osteoarthritis After Completing a 6-Week Positive Psychological Intervention or Neutral Control Program^a^

Outcomes	Positive Psychological Intervention				Neutral Control Program				Race × Program × Time Interaction^b^	
	Baseline	1 mo	3 mo	6 mo	Baseline	1 mo	3 mo	6 mo	χ^2^	*P* Value
Pain (WOMAC)^c^										
White									1.03	.79
No.	89	82	79	79	88	73	79	82		
Mean (SD)	45.2 (15.7)	42.4 (15.8)	40.1 (16.9)	39.2 (18.0)	45.1 (17.3)	42.3 (21.8)	40.8 (18.8)	39.0 (18.7)		
Change from baseline		−2.8	−5.2	−6.0		−2.8	−4.3	−6.1		
African American										
No.	90	74	76	77	90	76	74	71		
Mean (SD)	55.2 (16.7)	48.8 (20.8)	47.8 (20.6)	47.4 (23.0)	49.7 (18.6)	44.8 (20.1)	46.0 (19.2)	44.5 (20.9)		
Change from baseline		−6.4	−7.4	−7.8		−4.9	−3.7	−5.1		
Functional difficulty (WOMAC)^c^										
White									3.09	.38
No.	89	82	78	77	85	68	73	79		
Mean (SD)	43.6 (17.1)	40.1 (16.3)	39.9 (17.3)	40.6 (17.5)	44.2 (17.9)	40.0 (22.0)	40.5 (20.0)	39.1 (18.7)		
Change from baseline		−3.6	−3.7	−3.1		−4.1	−3.6	−5.1		
African American										
No.	88	74	75	75	89	74	74	69		
Mean (SD)	52.3 (17.4)	49.6 (22.1)	47.0 (20.1)	47.3 (21.9)	47.1 (19.1)	45.9 (19.7)	46.1 (19.4)	45.1 (21.0)		
Change from baseline		−2.7	−5.3	−5.0		−1.3	−1.1	−2.1		

Abbreviation: WOMAC, Western Ontario and McMaster Universities Osteoarthritis Index.

^a^ Means shown are unadjusted.

^b^ *P* values are based on χ^2^ tests of the 3-way interaction of program, race, and time from linear mixed models controlling for study site.

^c^ Higher scores indicate worse symptoms and negative change in scores indicates improvement.

### Secondary Outcomes: Affect Balance and Life Satisfaction

**Figure 2.**
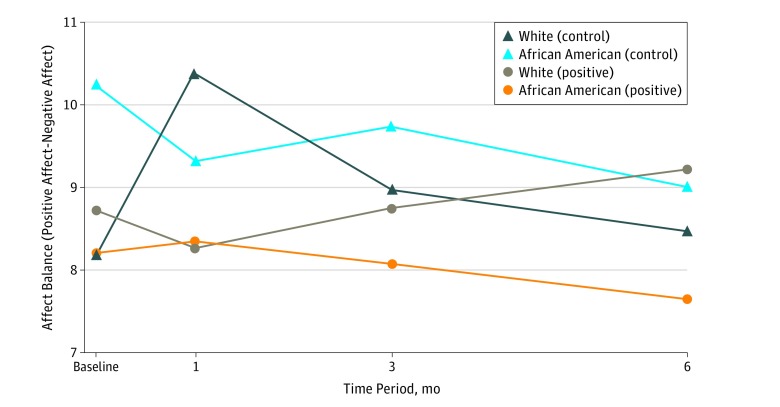
Mean Affect Balance by Race and Treatment Arm Over Time These means are provided to aid in interpretation of the significant interaction between treatment group, race, and time for affect balance (positive − negative affect scores), χ^2^_3_ = 8.64; *P* = .03. Among white participants, affect balance decreased from baseline to 1 month in the positive group, but increased over the same time in the control group. Among African American participants, affect balance decreased slightly at 1 month in the control group, and steadily declined over each time point in the positive group.

Mean (SD) affect balance at baseline was 8.8 (6.4), indicating more positive than negative affect overall. The 3-way interaction between treatment group, race, and time was significant (χ^2^_3_ = 8.64; *P* = .03). Examining the means indicated that among white patients affect balance decreased from baseline to 1 month in the positive group, but increased over the same time in the control group (Figure 2). Among African American patients, affect balance decreased slightly at 1 month in the control group, and steadily declined over each time point in the positive group.

Mean (SD) life satisfaction at baseline was 15.5 (5.1). The 3-way interaction between treatment group, race, and time was not significant (χ^2^_3_ = 1.79; *P* = .62). When race was removed, neither the treatment group × time interaction nor main effect of time was significant.

### Exploratory Subgroup Analyses

We explored 2 explanations for the lack of differences between treatment groups in the change in pain and functional difficulty—insufficient disease severity and nonadherence. For disease severity, we restricted analyses to participants with above-median baseline pain and functional difficulty, and to those with radiographic evidence of OA. Results in these subgroups were the same as those from the full sample.

For nonadherence, we tested the 3-way interaction between treatment group, race, and time, and the 2-way interaction between treatment group and time, in participants who reported completing 5 or more assigned activities. Results for pain and functional difficulty did not change. However, for life satisfaction the treatment group × time interaction was significant (χ^2^_3_ = 8.01; *P* = .05). The means indicated that, compared with baseline, life satisfaction decreased at 1 month and then rebounded back toward baseline in the positive group, whereas life satisfaction increased slightly at 1 month and decreased at later time points in the control group (positive, mean: 16.9 vs 16.0 vs 16.4 vs 16.6; control, mean: 15.2 vs 15.6 vs 15.3 vs 14.7 at baseline and 1, 3, and 6 months, respectively).

## Discussion

This randomized clinical trial was powered to detect racial differences in the effects of positive psychological interventions on chronic pain in older military veterans with knee OA. Although there were statistically significant reductions in pain and functional difficulty from baseline to 6 months, the differences were small and did not vary by treatment group or race. Affect balance and life satisfaction, core processes by which positive psychological interventions are thought to improve well-being, also did not show the predicted changes. In short, our study did not detect benefits of positive psychological interventions relative to a neutral control program for pain, functional difficulty, or measures of well-being in African American or white veterans with knee OA.

Our intervention may not have shown the hypothesized benefits owing to aspects of the patient population, study design, or the intervention itself. Our sample was older, more racially diverse, and more likely to be male compared with samples included in most prior positive psychological intervention studies. Our eligibility criteria allowed patients with a wide range of arthritis symptoms and those who were actively engaged in other pain treatments to enroll in the study. Although we erred on the side of inclusivity for pragmatic reasons and to increase generalizability, our broad criteria may have produced a sample with insufficient pain to show an effect of the intervention, or masked the response to the intervention by other treatments. These are unlikely explanations for our findings, however, given that the hypothesized effects did not occur in subgroups with more severe symptoms or radiologic evidence of OA, or after controlling for co-occurring pain treatments.

Additional pragmatic design choices make it difficult to know if the intervention would have had the expected effects under ideal circumstances. We did not assess outcomes during the intervention period to reduce patient burden and because interventions with only fleeting benefits are not sustainable in practice. We also compared our intervention with an active control condition to assess the active ingredients of positive psychological interventions and to account for alternative explanations such as motivation, placebo effects, or attention. Without a usual care control group, we do not know how the observed decreases in pain and functional difficulty compare with similar patients who did not participate in the study. Our measures also may not have been sufficiently sensitive to capture the effects of the intervention. There is wide within-person variability in patient-reported WOMAC pain and functional difficulty over time, especially among African American patients.^57^ Veterans may have derived benefit or satisfaction from the intervention that were not captured by our measures.

We conducted extensive pilot testing to adapt evidence-based positive psychological activities to the preferences and needs of veterans.^24^ The intervention showed acceptability and feasibility in pilot testing, and self-reported adherence to the program was reasonable in this trial. Nevertheless, the intervention did not show benefits for pain, functional difficulty, or well-being in this sample. Moreover, it showed subtle signs of backfiring on measures of well-being. For some patients, it is possible that activities in the intervention shed light on aspects of their lives that increased rather than decreased distress, such as reminding them of loved ones who have died, or the repetitiveness and isolation of their lives. Additional tailoring of individual activities, or including different activities that avoid such pitfalls, is needed for positive psychological interventions to be used effectively in this population.

The intervention also may have been ineffective because it did not focus explicitly on changing maladaptive pain-related emotions, thoughts, or behaviors, as do other psychological treatments for pain (eg, cognitive behavior therapy). Focusing on increasing positive affect, without addressing thoughts and behaviors that can worsen pain perception, may be insufficient to exert a meaningful shift in the central response to OA pain perception. For patients with chronic pain to reap the benefits of positive psychological interventions, it may be necessary to integrate principles from positive psychology into more comprehensive pain treatment regimens.

Our findings are surprising and disappointing in light of growing interest in applying positive psychological interventions in different populations with particular clinical conditions.^3,4,5,7,11,13,15,16,18^ Several studies describe how positive psychological interventions have been adapted for specific patient populations and delivery modalities, and demonstrate feasibility of such interventions in pilot studies.^11,12,13,14,15,16,17,18,20,21,22,23,24^ As one of the first completed large-scale randomized clinical trials with an active control group, this study does not demonstrate the benefits suggested by preliminary studies. Rather, it adds to a growing number of studies suggesting that effects of positive psychological interventions reported in early studies are smaller or nonexistent in later replications.^58,59,60^ In our pilot work, the positive psychological intervention showed medium to large effects on pain, difficulty functioning, and life satisfaction,^24^ none of which were maintained in the fully powered study. This underscores the imprecision of small pilot studies,^61^ and serves as a cautionary tale for moving forward with implementation of practices for which only preliminary evidence is available. Our study also aligns with a study showing that an active control program outperformed a positive psychological intervention among patients hospitalized with suicidal thoughts, suggesting that positive psychological interventions are not a panacea for all patient populations.^62^

This study was motivated by the need for effective, nonpharmacological treatments for alleviating OA pain and functional difficulties. Multiple psychological approaches to pain treatment (eg, cognitive behavioral therapy and mindfulness-based stress reduction) have been developed and tested for patients with chronic pain,^63^ but high quality evidence demonstrating the effectiveness of such approaches for patients with knee OA is lacking. A recent review examining evidence for the impact of psychological interventions on pain concluded that there is a dearth of strong empirical evidence that psychological treatments for pain management are effective.^64^ While the association of modifiable cognitions and behaviors with OA pain and functional difficulty (eg, pain catastrophizing, depression, and pain coping strategies) is well documented,^65,66,67,68^ interventions that target psychological or behavioral pain mechanisms and produce large improvements in pain outcomes remain elusive.

### Limitations

Our study sample was limited to patients with knee OA from 2 VA medical centers, thereby limiting the generalizability of our findings to patients with other chronic pain conditions, nonveterans, or veterans being treated at other VA or non-VA facilities. Although we excluded patients with inflammatory arthritis conditions other than OA, we did not assess for pain conditions other than arthritis, so patients could have had pain from multiple illnesses. Our adherence and outcomes were self-reported and thus vulnerable to measurement bias. As noted, the omission of a usual care group makes it unclear how the changes we observed compare with similar patients not enrolled in the study.

## Conclusions

This large 2-site randomized clinical trial of a positive psychological intervention for chronic pain fills important gaps in the literature by testing the use of such interventions in older veterans with chronic pain, testing for racial differences in response to such interventions, and comparing their long-term effects with those of a strong control group. Unfortunately, the results do not support the use of positive psychological interventions as a stand-alone psychological treatment for pain among white or African American veterans with knee OA. Adaptations are needed to identify specific positive psychological intervention components that resonate with this population, and the potential additive effect of incorporating positive psychological interventions into comprehensive pain treatment regimens should be considered.

## Notes

Supplement 1. **Trial Protocol**

Supplement 2. **eTable 1.** Reasons for Ineligibility **eTable 2.** Reasons for Missing Data Points **eTable 3.** Reasons for Withdrawing From the Study **eTable 4.** Adherence to Positive and Control Programs by Race **eTable 5.** Ratings of Positive and Control Programs by Race
