# Supplementary material for: Effect of a Positive Psychological Intervention on Pain and Functional Difficulty Among Adults With Osteoarthritis: A Randomized Clinical Trial
Source: JAMA Netw Open. 2018 Sep 21;1(5):e182533. doi: 10.1001/jamanetworkopen.2018.2533 (PMC6324470; doi:10.1001/jamanetworkopen.2018.2533)
Supplement: Supplement 1. — Trial Protocol [file jamanetwopen-1-e182533-s001.pdf]

ClinicalTrials.gov Protocol Registration and Results System (PRS) Receipt
Release Date: July 9, 2018

ClinicalTrials.gov ID: NCT02223858

Study Identification

Unique Protocol ID: IIR 13-080

Brief Title: Staying Positive With Arthritis Study (SPA)

Official Title: Staying Positive: An Intervention to Reduce Osteoarthritis Pain Disparities

Secondary IDs:

Study Status

Record Verification: July 2018

Overall Status: Completed

Study Start: July 13, 2015 [Actual]

Primary Completion: November 9, 2017 [Actual]

Study Completion: November 9, 2017 [Actual]

Sponsor/Collaborators

Sponsor: VA Office of Research and Development

Responsible Party: Sponsor

Collaborators:

Oversight

U.S. FDA-regulated Drug: No

U.S. FDA-regulated Device: No

Unapproved/Uncleared Device: No

U.S. FDA IND/IDE: No

Human Subjects Review: Board Status: Approved
Approval Number: 14-08
Board Name: VA Central IRB
Board Affiliation: Department of Veterans Affairs
Phone: (202) 443-5660
Email: Christie.O'Brien@va.gov
Address:

VA Greater Los Angeles Healthcare System, West Los Angeles, CA
11301 Wilshire Boulevard

West Los Angeles, CA 90073
Country: USA

Data Monitoring: No

Study Description

Brief Summary: Arthritis is a painful, disabling condition that disproportionately affects African Americans. Existing arthritis treatments yield only small to moderate improvements in pain and are not effective at reducing racial disparities in arthritis pain. According to the biopsychosocial model of pain, there is a need for novel interventions that target psychosocial factors associated with arthritis outcomes and disparities in outcomes. Evidence from the field of psychology suggests that an intervention designed to develop a positive mindset has the potential to improve pain and functioning and reduce racial disparities in patients with arthritis. Interventions to foster a positive mindset have been developed for clinical patient populations but have not yet been fully tested in patients with arthritis or in Veterans, nor have their effects on racial differences in clinical outcomes been examined. This study will address these gaps by testing the impact of an evidence-based positive activities intervention on pain and functioning in African American and White Veterans with knee arthritis.

Detailed Description: Background: Arthritis is a prevalent and disabling source of chronic pain for which African Americans (AAs) bear a disproportionate burden. The purpose of this study is to test a patient-centered, non-invasive intervention to improve pain outcomes and reduce disparities in AA and White Veterans with knee arthritis. The intervention is designed to help Veterans develop a positive mindset, the health benefits of which are well-documented.

Objectives: The primary aim of this study is to evaluate the impact of a positive intervention on pain and physical functioning in AA and White Veterans with knee arthritis through a randomized, controlled, clinical trial. It is hypothesized that patients randomized to a positive activities (PA) intervention will experience improved pain and functioning compared to patients randomized to an attention control (AC) program, and that these improvements will be larger for AA than for WH Veterans. The secondary aim of this study is to identify variables that mediate the effects of the PA intervention on pain and functioning. It is hypothesized that the effects of the PA intervention will be mediated by psychosocial variables known to be associated with arthritis outcomes or racial differences in arthritis outcomes (e.g., depression, self-efficacy, pain coping, perceived discrimination).

Methods: A randomized, controlled, 2-arm design will be used to compare the effects of a 6-week PA intervention with that of an AC program on pain and functioning at 1, 3, and 6-months post-intervention among AA and WH Veterans with knee arthritis. Approximately 180 AA and 180 WH primary care patients with knee pain symptoms consistent with arthritis will be recruited from participating VA medical centers following the original protocol. [Due to accelerated recruitment of the original target sample, up to 240 additional primary care patients with knee pain symptoms consistent with OA will be recruited from participating VA medical centers using inclusion criteria that take into account original ICD-9 codes and their corresponding ICD-10 codes. The additional patients (including some men and some women, as resources allow) will be recruited to increase power to detect sex differences in secondary analyses after the primary aims of the study have been achieved using the original cohort.] Eligible participants will complete an in-person baseline assessment of study outcomes, mediators, and control variables and be randomized to a 6-week PA or AC program. The PA program consists of completing 6 at-home activities (1 per week) that have been shown to increase positivity. The AC program consists of 6 affectively neutral activities. Both

groups will receive weekly telephone calls from trained interventionists to clarify instructions for the next week's activity and assess completion of the previous week's activity. Outcomes and proposed mediating variables will be assessed via telephone surveys at 1 month, 3 months, and 6 months post-intervention. Study outcomes include self-reported pain and physical functioning as measured by the Western Ontario MacMaster Index. Hypothesized mediators include depressive symptoms, positive/negative affect, satisfaction with life, arthritis self-efficacy, pain coping, pain catastrophizing, perceived discrimination, global stress, and social support. The intervention impact over time and by race (primary aim) will be tested using linear mixed models that allow repeated measures on the continuous outcomes for each participant and assess change in outcomes over time. A multiple mediator bootstrap approach to assess whether the effect of the intervention is mediated by the hypothesized mediators.

Conditions

Conditions: Arthritis

Keywords: Health Status Disparities
Arthritis
Psychology
Pain Management
Affect

Study Design

Study Type: Interventional

Primary Purpose: Health Services Research

Study Phase: N/A

Interventional Study Model: Factorial Assignment
Randomization will be at the patient level, stratified by study site and patient race, with a 1:1 allocation using random block sizes of 2, 4, 6, or 8.

Number of Arms: 2

Masking: Double (Participant, Outcomes Assessor)
The statistician will seal PA and AC program workbooks in envelopes following the randomization scheme. To blind participants and staff during the baseline assessment, staff will take the next sealed envelope in the sequence to each baseline visit, to be opened after a patient has consented and completed the baseline assessment. The study staff that complete the baseline visits will be unblinded once a participant's envelope is opened; participants will not be told whether they are in the PA or AC program. Staff who conduct the weekly intervention calls in the 6 weeks following the baseline visit will also be unblinded. To maintain blinding for the collection of outcome measures, study staff members who did not complete the baseline visit or any weekly calls during the 6-week program period for a given participant will collect the 1, 3, and 6-month follow-up assessments.

Allocation: Randomized

Enrollment: 517 [Actual]

Arms and Interventions

Arms	Assigned Interventions
Experimental: Positive Activities (PA)<br>Patients in the PA arm will be asked to complete a 6-week program consisting of 6 at-home activities (1 completed per week) that have been shown to increase positivity. PA activities will be delivered using a combination of activity booklets and oral instructions provided during weekly telephone calls from trained interventionists. Interventionist will orient participants to the booklets and review the first activity at the end of an in-person baseline visit. The booklet contains all instructions patients need to complete the full program. However, the interventionist will conduct weekly telephone calls to provide additional support patients may need. During these calls, the interventionist will assess whether participants completed the previous week's activity, review instructions for the next week's activity, and help participants trouble-shoot anticipated barriers to completing the next activity.	Behavioral: Positive Activities (PA) Program<br>This is a 6-week program consisting of at-home activities (1 completed per week) that have been shown to increase positivity. Activities will be delivered using a combination of activity booklets and oral instructions provided during weekly telephone calls from trained interventionists. Interventionist will orient participants to the booklets and review the first activity at the end of an in-person baseline visit. The booklet contains all instructions patients need to complete the full program. However, the interventionist will conduct weekly telephone calls to provide additional support patients may need. During these calls, the interventionist will assess whether participants completed the previous week's activity, review instructions for the next week's activity, and help participants trouble-shoot anticipated barriers to completing the next activity.
Active Comparator: Attention Control (AC)<br>Patients in the AC arm will be asked to complete a 6-week program consisting of at-home activities (1 completed per week) that are based on affectively neutral activities from control conditions in prior studies of PA interventions. Activities will be delivered using a combination of activity booklets and oral instructions provided during weekly telephone calls from trained interventionists. Interventionist will orient participants to the booklets and review the first activity at the end of an in-person baseline visit. The booklet contains all instructions patients need to complete the full program. However, the interventionist will conduct weekly telephone calls to provide additional support patients may need. During these calls, the interventionist will assess whether participants completed the previous week's activity, review instructions for the next week's activity, and help participants trouble-shoot anticipated barriers to completing the next activity.	Behavioral: Attention Control (AC) Program<br>This is a 6-week program consisting of at-home activities (1 completed per week) that are based on affectively neutral activities from control conditions in prior studies of positive activities interventions. Activities will be delivered using a combination of activity booklets and oral instructions provided during weekly telephone calls from trained interventionists. Interventionist will orient participants to the booklets and review the first activity at the end of an in-person baseline visit. The booklet contains all instructions patients need to complete the full program. However, the interventionist will conduct weekly telephone calls to provide additional support patients may need. During these calls, the interventionist will assess whether participants completed the previous week's activity, review instructions for the next week's activity, and help participants trouble-shoot anticipated barriers to completing the next activity.

Outcome Measures

Primary Outcome Measure:

1. Change in self-reported pain from baseline to 1, 3, and 6 months post-intervention
   Pain subscale of the Western Ontario MacMaster (WOMAC) Index

   [Time Frame: Baseline to 6 months post-intervention]

2. Change in self-reported physical functioning from baseline to 1, 3, and 6 months post-intervention
   Difficulty with physical functioning subscale of the Western Ontario MacMaster (WOMAC) Index

   [Time Frame: Baseline to 6 months post-intervention]

Other Pre-specified Outcome Measures:

3. Change in patient global assessment of pain from baseline to 1, 3, and 6 months post-intervention
   Self-reported global assessment of pain in the last week using a numeric rating scale

[Time Frame: Baseline to 6 months post-intervention]

Eligibility

Minimum Age: 50 Years

Maximum Age:

Sex: All

Gender Based: No

Accepts Healthy Volunteers: No

Criteria: Inclusion Criteria:

The target population will be African American (AA) and White (WH) Veterans with symptomatic knee arthritis. Specific inclusion criteria include:

- Age 50 years or older
- Receive primary care at a participating study site
- Self-report as non-Hispanic black/AA or non-Hispanic WH
- Frequent, symptomatic knee pain identified using questions from the OA Initiative
- Pain level of 4 or higher on a 0-10 numeric rating scale
- Can speak, read, and write in English

Exclusion Criteria:

Patients will be excluded if they:

- Report serious problems with hearing, eyesight, or memory
- Report having been diagnosed any type of arthritis other than osteoarthritis or degenerative arthritis
- Report that they have been treated for cancer in the last 3 years
- Report having had a steroid injection into one or both knees in the past 3 months
- Report having had a knee replacement into one or both knees in the past 3 months
- Report having plans to have a knee replacement in one or both knees in the next 6 months
- Report that there is a reason they cannot complete the study procedures, which include telephone calls and program activities that involve reading and writing
- Do not have a telephone number where they can receive telephone calls from research staff
- Screen positive for cognitive impairment

Contacts/Locations

Central Contact Person: Leslie R Hausmann, PhD
Telephone: (412) 360-2112
Email: leslie.hausmann@va.gov

Central Contact Backup: Genna M Mauro, BS
Telephone: (412) 360-2131
Email: Genna.Mauro@va.gov

Study Officials: Leslie R. M. Hausmann, PhD
Study Principal Investigator
VA Pittsburgh Healthcare System University Drive Division, Pittsburgh, PA

Locations: United States, Pennsylvania
VA Pittsburgh Healthcare System University Drive Division, Pittsburgh, PA
Pittsburgh, Pennsylvania, United States, 15240
Contact: Leslie R Hausmann, PhD (412) 360-2112 leslie.hausmann@va.gov
Contact: Genna M Mauro, BS (412) 360-2131 Genna.Mauro@va.gov
Sub-Investigator: Acacia Parks, PhD
Sub-Investigator: Debra K. Weiner, MD
Principal Investigator: Leslie R. M. Hausmann, PhD

Corporal Michael J. Crescenz VA Medical Center, Philadelphia, PA
Philadelphia, Pennsylvania, United States, 19104
Contact: Said A Ibrahim, MD MPH MBA 215-823-5847 said.ibrahim2@va.gov
Contact: Aliya S Collier (215) 823-5800 Ext. 7913 Aliya.Collier@va.gov
Sub-Investigator: Said A. Ibrahim, MD MPH MBA

IPDSharing

Plan to Share IPD: No

References

Citations: Hausmann LRM, Ibrahim SA, Kwoh CK, Youk A, Obrosky DS, Weiner DK, Vina E, Gallagher RM, Mauro GT, Parks A. Rationale and design of the Staying Positive with Arthritis (SPA) Study: A randomized controlled trial testing the impact of a positive psychology intervention on racial disparities in pain. Contemp Clin Trials. 2018 Jan;64:243-253. doi: 10.1016/j.cct.2017.09.001. Epub 2017 Sep 8. PubMed 28893676

Hausmann LRM, Youk A, Kwoh CK, Ibrahim SA, Hannon MJ, Weiner DK, Gallagher RM, Parks A. Testing a Positive Psychological Intervention for Osteoarthritis. Pain Med. 2017 Oct 1;18(10):1908-1920. doi: 10.1093/pm/pnx141. PubMed 29044408

Links:

Available IPD/Information:
