# Supplementary material for: Effect of a Positive Psychological Intervention on Pain and Functional Difficulty Among Adults With Osteoarthritis: A Randomized Clinical Trial
Source: JAMA Netw Open. 2018 Sep 21;1(5):e182533. doi: 10.1001/jamanetworkopen.2018.2533 (PMC6324470; doi:10.1001/jamanetworkopen.2018.2533)
Supplement: Supplement 2. — eTable 1. Reasons for Ineligibility eTable 2. Reasons for Missing Data Points eTable 3. Reasons for Withdrawing From the Study eTable 4. Adherence to Positive and Control Programs by Race eTable 5. Ratings of Positive and Control Programs by Race [file jamanetwopen-1-e182533-s002.pdf]

Supplementary Online Content

Hausmann LRM, Youk A, Kwoh CK, et al. Effect of a positive psychological intervention on pain and functional difficulty among adults with osteoarthritis: a randomized clinical trial. *JAMA Netw Open*. 2018;1(5):e182533. doi:10.1001/jamanetworkopen.2018.2533

eTable 1. Reasons for Ineligibility
eTable 2. Reasons for Missing Data Points
eTable 3. Reasons for Withdrawing From the Study
eTable 4. Adherence to Positive and Control Programs by Race
eTable 5. Ratings of Positive and Control Programs by Race

This supplementary material has been provided by the authors to give readers additional information about their work.

© 2018 Hausmann LRM et al. *JAMA Network Open*.

eTable 1. Reasons for Ineligibility

**Reasons**	**No. (of 351)***
Under 50 years old	2
Does not receive primary care at study site	30
Race other than non-Hispanic white or African American	29
Infrequent knee pain	83
Pain rating < 4 on 0-10 scale	77
Self-reported serious problems with hearing or eyesight	15
Diagnosed with any type of arthritis other than OA or degenerative arthritis	77
Treated for cancer in the last three years	73
Had a steroid injection into one or both knees in the past 3 months	50
Had a knee replacement into one or both knees in the past 3 months	3
Plan to have a knee replacement in one or both knees in the next 6 months	15
Self-reported inability to complete the study telephone calls and program activities that involve reading and writing	15
Lack of a reliable telephone number	3
Answering 2 or more items incorrectly on a 6-item screener for cognitive impairment	36
Deemed unable to participate by interviewer**	4

*Numbers will not add to 351 because individuals could be ineligible for multiple reasons.

**Scheduled for shot (n=1), knee pain due to an accident (n=1), difficulty completing screening survey (n=2)

© 2018 Hausmann LRM et al. *JAMA Network Open*.

eTable 2. Reasons for Missing Data Points

		White		African American	
**Time Point**	**Reasons**	**Control**	**Positive**	**Control**	**Positive**
Week 1	No answer	5	3	4	3
	Refused	2	3	3	6
	Withdrawn*	0	0	1	1
	Other	1	0	0	0
Week 2	No answer	8	2	5	7
	Refused	2	7	6	7
	Withdrawn*	1	1	1	2
	Other	2	0	0	0
Week 3	No answer	5	4	11	9
	Refused	2	4	4	11
	Withdrawn*	2	2	1	2
	Other	1	0	0	0
Week 4	No answer	5	5	9	7
	Refused	6	8	4	12
	Withdrawn*	2	2	2	3
	Other	1	0	0	0
Week 5	No answer	9	6	6	9
	Refused	2	8	5	9
	Withdrawn*	2	2	2	3
	Other	0	0	0	0
Week 6	No answer	6	7	7	4
	Refused	3	4	3	8
	Withdrawn*	2	2	2	3
	Other	0	1	0	0
1-month follow-up	No answer	7	1	7	4
	Refused	5	4	5	7
	Withdrawn*	3	3	2	4
	Other	1	0	0	1
3-month follow-up	No answer	3	3	7	6
	Refused	4	4	7	3
	Withdrawn*	3	4	2	4
	Other	0	0	0	1
6-month follow-up	No answer	3	3	8	3
	Refused	1	4	8	5
	Withdrawn*	3	4	2	4
	Other	0	0	1	1

*See eTable 3 for reasons for withdrawing from the study.

© 2018 Hausmann LRM et al. *JAMA Network Open*.

eTable 3. Reasons for Withdrawing From the Study

	White		African American	
	**Control**	**Positive**	**Control**	**Positive**
Total number withdrawn	3	4	2	4
Reasons for withdrawing*				
Did not think activities would help	1	2	2	3
Too busy	1	1	1	1
Activities too difficult	0	0	0	1
Inadequate payments	0	0	0	1
Unrelated health problems	1	0	0	0
No reason given	0	1	0	1

*Participants could withdraw for multiple reasons.

© 2018 Hausmann LRM et al. *JAMA Network Open*.

eTable 4. Adherence to Positive and Control Programs by Race

**Adherence, n (%)***	**Total (n=360)**	**Treatment Group**			**Participant Race**		
		**Positive (n=180)**	**Control (n=180)**	**Test Statistic**	**White (n=180)**	**African American (n=180)**	**Test Statistic**
Completed at least 5 of 6 calls	287 (79.7)	142 (78.9)	145 (80.6)	$\chi^2(1)=0.16$, p=0.69	146 (81.1)	141 (78.3)	$\chi^2(1)=0.44$, p=0.51
Entirely or partially completed at least 5 of 6 activities	234 65.0)	121 (67.2)	113 (62.8)	$\chi^2(1)=.79$, p=0.37	126 (70.0)	108 (60.0)	$\chi^2(1)=4.00$, p=0.05

*****Participants who did not complete a weekly call were counted as not completing that week's activity. P-values comparing adherence rates are from logistic regression models. Positive vs. neutral and White vs. African American p-values are from main effect models that include study site. There were no significant Treatment Group by Race interactions (data not shown). See eTable 2 for weekly adherence rates.

© 2018 Hausmann LRM et al. *JAMA Network Open*.

eTable 5. Ratings to Positive and Control Programs by Race

Ratings of Completed Weekly Activities, mean (SD)*	Total (n=1620)	Treatment Group			Participant Race		
		Positive (n=814)	Control (n=806)	Test Statistic	White (n=839)	African American (n=781)	Test Statistic
Perceived benefit	5.58 (1.52)	5.77 (1.32)	5.39 (1.68)	$\chi^2(1)=10.27$, $p=0.001$	5.54 (1.52)	5.62 (1.52)	$\chi^2(1)=0.84$, $p=0.36$
Perceived enjoyment	5.62 (1.55)	5.91 (1.30)	5.33 (1.72)	$\chi^2(1)=22.17$, $p<0.001$	5.60 (1.53)	5.64 (1.57)	$\chi^2(1)=0.17$, $p=0.68$
Perceived difficulty	2.24 (1.83)	2.26 (1.80)	2.23 (1.87)	$\chi^2(1)=0.00$, $p=0.95$	2.24 (1.79)	2.25 (1.87)	$\chi^2(1)=0.00$, $p=0.96$

*Activities were rated on 7-point Likert scales (1=not at all to 7=extremely). P-values comparing activity ratings are from mixed-effects linear regression models using maximum likelihood estimation. The analyses included multiple observations per patient, with observation weeks only analyzed for those recalling the correct activity for the week (1620 observation weeks nested within 333 participants). Positive vs. neutral and White vs. African American p-values are from main effect models that included study site. There were no significant Treatment Group by Race interactions (data not shown).

© 2018 Hausmann LRM et al. *JAMA Network Open*.
